# Self-powered ZnS Nanotubes/Ag Nanowires MSM UV Photodetector with High On/Off Ratio and Fast Response Speed

**DOI:** 10.1038/s41598-017-05176-5

**Published:** 2017-07-07

**Authors:** Qinwei An, Xianquan Meng, Ke Xiong, Yunlei Qiu

**Affiliations:** 1 School of Physics and Technology, Wuhan University, Wuhan, Hubei 430072 PR China; 2 Hubei Nuclear Solid Physics Key Laboratory, Hubei, 430072 PR China

## Abstract

In this study, we design and demonstrate a novel type of self-powered UV photodetectors (PDs) using single-crystalline ZnS nanotubes (NTs) as the photodetecting layer and Ag nanowires (NWs) network as transparent electrodes. The self-powered UV PDs with asymmetric metal-semiconductor-metal (MSM) structure exhibit attractive photovoltaic characteristic at 0 V bias. Device performance analysis reveals that the as-assembled PDs have a high on/off ratio of 19173 and a fast response speed (τ_r_ = 0.09 s, τ_f_ = 0.07 s) without any external bias. These values are even higher than that of ZnS nanostructures- and ZnS heterostructure-based PDs at a large bias voltage. Besides, its UV sensivity, responsivity and detectivity at self-powered mode can reach as high as 19172, 2.56 A/W and 1.67 × 10^10^ cm Hz^1/2^ W^−1^, respectively. In addition, the photosensing performance of the self-powered UV PDs is studied in different ambient conditions (e.g., in air and vacuum). Moreover, a physical model based on band energy theory is proposed to explain the origin of the self-driven photoresponse characteristic in our device. The totality of the above study signifies that the present self-powered ZnS NTs-based UV nano-photodetector may have promising application in future self-powered optoelectronic devices and integrated systems.

## Introduction

The UV PDs devices have received considerable attention owing to their wide range of commercial and scientific applications, such as biological and chemical analysis, binary switches in imaging techniques, flame sensing, communications, missile detection, astronomical studies^1–4^. In these UV PDs, an external bias is indeed required as the driving force to prevent the recombination of photogenerated electron-hole pairs.The need of an external power supply in traditional UV PDs, not only increases the system’s size, cost and energy consumption, but also limits their applicability in long-term constant UV monitoring in unmanned hazardous environments. Therefore, low power consumption and high performance UV photodetectors are urgently needed. To date, a number of innovative UV photodetectors have been successfully fabricated to function without external power sources, namely, they are self-powered (SP) PDs. For example, TiO_2_^5^, ZnO^6–8^, SnO_2_^9, 10^, SbSl^11^ and other wide-bandgap semiconductors have been successfully applied to create various types of self-powered UV photodetectors, in which the self-powered UV photodetectors architectures mainly include p-n junctions photodiode^5–8^ and Schottky junctions diodes^11–13^.

The p-n junctions photodiode based self-powered UV PDs are constructed using a pair of p- and n-type semiconductors with a wide band gap^14, 15^. It is demonstrated that the p-n junction photodiode-based self-powered UV PDs usually possesses a good spectral selection (visible-blind) and a fast photoresponse^7, 8^. However, lower electron recombination results in a lower saturation current density of the device, which in turn leads to a larger open circuit voltage (V_oc_)^15^. In addition, the performance comes at a price; the high temperatures and ultraclean p- and n-type materials make this kind of self-powered UV-PDs very expensive. The Schottky barrier-based self-powered UV PDs, fabricated by depositing a metal with high work function on n-type semiconductor, could be a lower-cost alternative to p-n junctions. When illuminated by UV light (photon energy is larger than the E_g_ of the used semiconductor), the electron-hole pairs are generated and quickly separated due to the built-in electric field induced by Schottky barrier, showing a obvious photovoltaic (PV) effect. Schottky barrier-based self-powered UV PDs often show extremely high photosensitivity ((I_photo_−I_dark_)/I_dark_) and fast photoresponse^11, 16^. However, the Schottky barrier that impedes the recombination of electrons at anode is relatively small, resulting in a saturation current density that is typically much larger than that of p-n junction diodes^15^, which makes the responsivity and detectivity are lower than that of the p-n junction photodiode-based self-powered UV PDs. Very recently, a novel self-powered UV PDs have been developed using an asymmetric metal-semiconductor-metal (MSM) structure^17^. In comparison of the conventional photovoltaic PDs, the novel self-powered UV PDs have a higher responsivity and UV/VIS rejection ratio, and much faster photoresponse speed. More importantly, this design provides a new route to realize self-powered photodetectors in terms of ease of construction, low fabrication cost, and scalability.

Nanotube with unique advantages and some excellent properties has shown great potential in realizing high-performance optoelectronic devices. For example, the diffusion paths of migrating charge carriers in hollow nanotube are typically an order of magnitude lower (tens compared to hundreds of nanometre), which further significantly influence electron-hole lifetimes and lead to higher efficiency of charge separation^18, 19^. Besides, the hollow nanotubes with larger surface-to-volume ratios and superior surface area than other one dimensional (1D) nanomaterials provide more space for the light reactions and gas molecules adsorption in the photo- and gas-sensor devices^18, 20^. Such architectures are expected to stimulate materials’ innovative utilization and fabricate faster response speed optoelectronic devices, e.g. electrochemistry, lithium ion batteries, supercapacitor, sensing, photocatalysis, dye-sensitized solar cells and biomedical devices^18–25^. Recently, ZnO nanotubes and TiO_2_ nanotubes have been used to fabricate photodetectors. The photodetectors based on ZnO nanotubes show that the photocurrent (4.82 × 10^−7^A) can be enhanced compared to that (0.571 × 10^−7^ A) based on ZnO NWs^25^. In our previous paper, we reported an entirely novel type of high-sensitivity photodetector using single-crystalline CdS NTs as active layer. In particular, the device exhibited enhanced sensitivity (4016) and larger turn-on voltages (0.3 V) compared with the sensitivity (741) and turn-on (0.21 V) voltage of CdS nanowires photodetector with the same device construction strategies^20^. However, to the best of our knowledge, there is no report about ZnS nanotubes used as photodetecting materials in UV photodetectors device.

In the present work, large amounts of high-quality tubular ZnS nanotubes have been obtained via a one-step thermal evaporation process without using any templates, with which as photodetecting materials high performance self-powered UV PDs with large on/off ratio, high response speed and low energy consumption have been designed and fabricated. Besides, the fully nanostructured PDs concept using the ZnS NTs network as active layer and Ag NWs network as electrodes allows the fabrication of devices through economic and wide scale available methods and display great potential in large area electronics that are flexible and transparent^26–29^. The performance analysis of the as-fabricated device demonstrates a high on/off ratio of 19173 and a fast response speed (τ_r_ = 0.09 s, τ_f_ = 0.07 s) without any external bias. These values are even higher than that of ZnS nanostructures- and ZnS heterostructure-based PDs at a large bias voltage^30–36^. Its UV photosensitivity, responsivity and detectivity at self-powered mode can reach as high as 19172, 2.56 A/W and 1.67 × 10^10^ cm Hz^1/2^ W^−1^, respectively. To the best of our knowledge, this is the first time ZnS nanotubes are used as UV photodetecting materials in self-powered UV PDs showing high performance while requiring no energy to operate. In addition, the photosensing performance of the self-powered ZnS NTs-based asymmetric MSM UV PDs is studied in different ambient conditions (e.g., in air and vacuum).

## Results and Discussion

**Figure 1:**
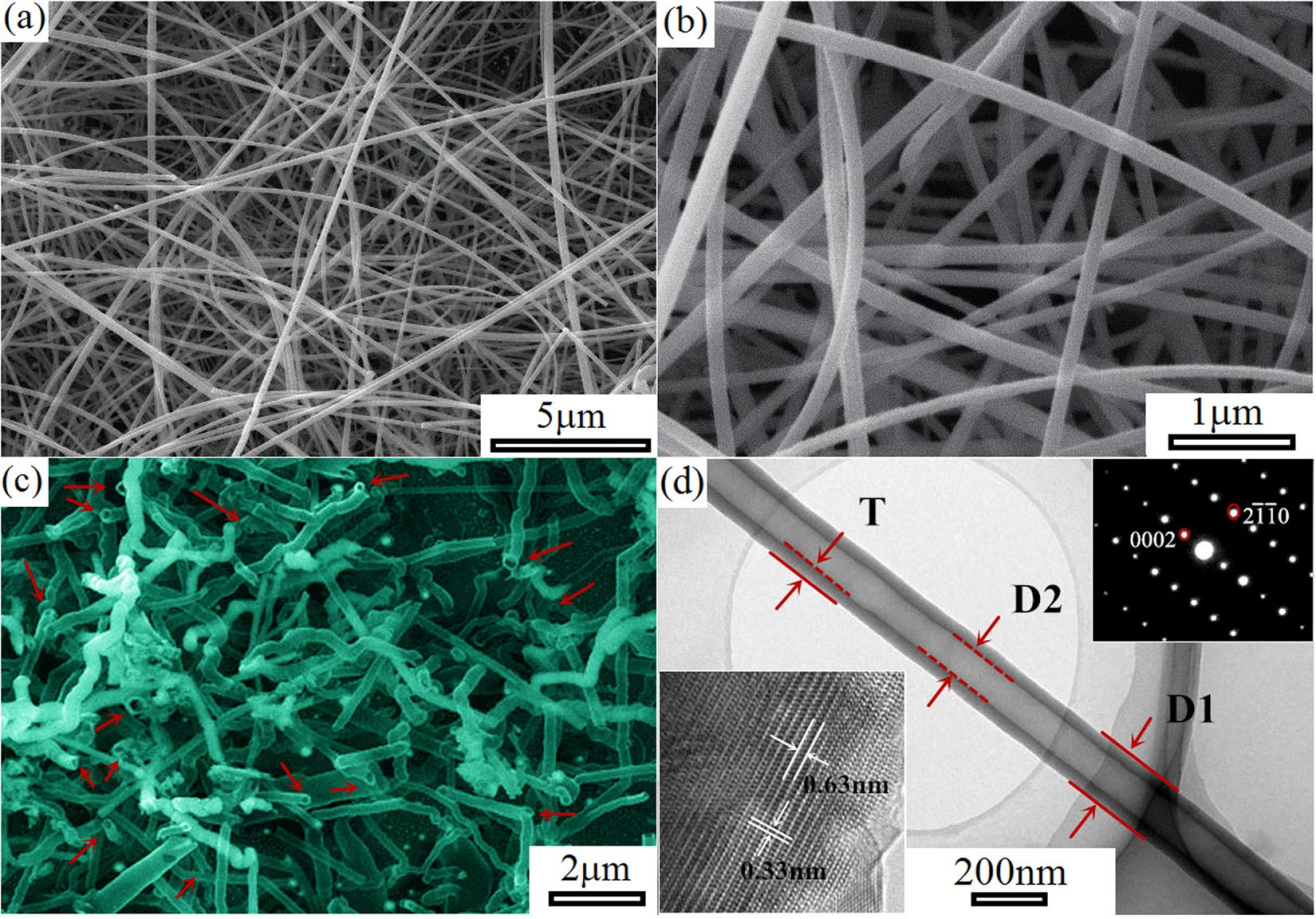
(**a**) Low-magnification SEM image and (**b**) high-magnification SEM image of ZnS nanotubes. (**c**) SEM image of broken ZnS nanotubes, the tube openings marked by red arrows. (**d**) TEM image of individual ZnS nanotube, inset shows HR-TEM image and selected area electron diffraction (SAED) patterns of ZnS nanotube.

The morphology and structural characterizations of ZnS nanotubes are summarized in Fig. 1. The typical scanning electron microscopy (SEM) image of ZnS nanotubes (Fig. 1a) demonstrates a high yield of ZnS nanotubes can be obtained by thermal evaporation of ZnS powder, and their lengths range from tens of micrometers to several hundreds of micrometers. The high-magnification SEM image of ZnS nanotubes in Fig. 1b reveals that the average outer tube diameter of ZnS nanotubes is ~150 nm. As shown in Fig. 1c, the SEM image of broken nanotubes, which are prepared by using knife blade to remove most of ZnS nanotubes from the growth substrate, directly confirms the hollow structures of nanotubes. In addition, the tube openings marked by red arrows can be directly observed from the top-opened tubular morphology of these broken nanotubes. Figure 1d shows the TEM image of an individual ZnS nanotubes. The ZnS nanotube is uniform in diameters and thickness, and the typical outer diameter, inter diameter and wall thickness of the ZnS nanotube indicated with **D1**, **D2** and **T** is about 156, 73 and 42 nm, respectively. The insets show the HR-TEM image and the SAED pattern of the ZnS nanotube, the marked inter-planar d-spacings of 0.33 and 0.63 nm corresponds well with the (2-1-10) and (0002) lattice planes of wurtzite ZnS, respectively. The SAED pattern demonstrates the single crystalline and defect-free structure of the ZnS nanotube. In our previous research, the tubular growth mechanism of the ZnS nanotubes has been discussed in detail based on experimental results^37^.

**Figure 2:**
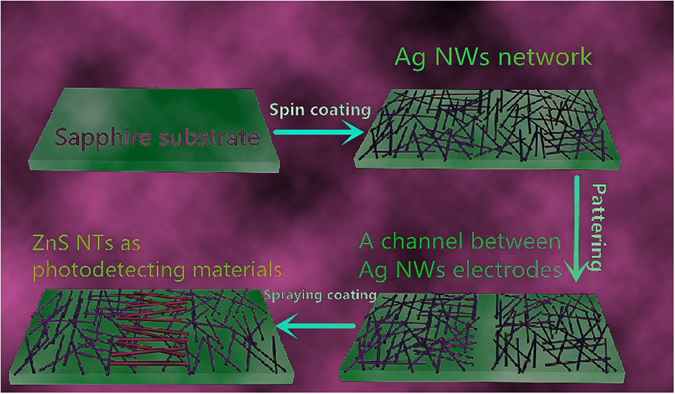
Schematic illustration of fabrication procedures for the nanostructured Ag NWs/ZnS NTs/Ag NWs (MSM) photodetectors.

Figure 2 illustrates the detailed procedures for the fabrication of the nanostructured Ag NWs/ZnS NTs/Ag NWs (MSM) PDs device. Firstly, transparent and conducting Ag NWs network was prepared on the sapphire substrate by spin coating. The Ag NWs network with transmittance and sheet resistance of 87% and 64 Ω/sq, respectively, was used as the transparent electrodes of the PDs device. To deposit ZnS nanotubes between the drain (D) and source (S) Ag NWs electrodes as photodetecting materials, a typical channel of ~40 μm between the D and S Ag NWs electrodes was provided. Through repeated spray coating, ZnS nanotubes networks were formed between the S/D Ag NWs electrodes.

**Figure 3:**
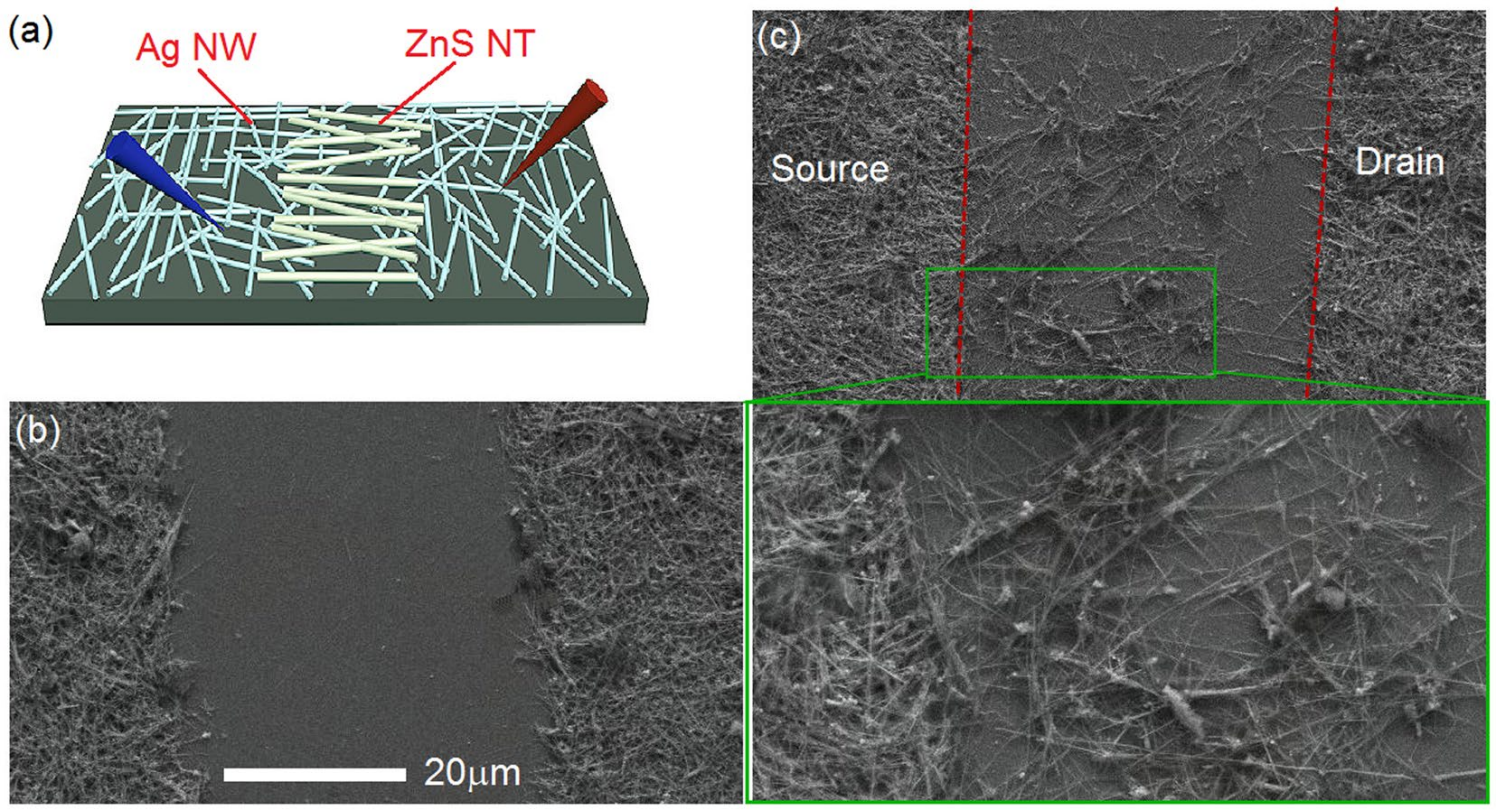
(**a**) Schematic view of the nanostructured Ag NWs/ZnS NTs/Ag NWs (MSM) PDs device. (**b**) SEM image of a channel between source and drain Ag NWs electrodes, which is free from Ag NWs prior to the ZnS NTs spray coating. (**c**) SEM image of the nanostructured MSM PDs device, the interface between Ag NWs and ZnS NTs are marked by red dashed lines. Inset shows the high-magnification SEM image of the interface between source Ag NWs electrode and ZnS NTs.

The I–V measurements of the nanostructured MSM PDs device are performed by using a two-probe method as schematically illustrated in Fig. 3a. Figure 3b presents the typical channel of ~40 μm between the D and S Ag NWs electrodes, which is free from Ag NWs prior to the ZnS NTs spray coating. The SEM image of the MSM PDs shown in Fig. 3c clearly confirms that the single-crystalline ZnS nanotubes are assembled into the channel between the S and D Ag NWs electrodes, and the interfaces between Ag NWs and ZnS NTs of the PDs device are clearly seen and marked by red dashed lines. The inset of Fig. 3c displays the corresponding magnified SEM image of the rectangle region as marked in Fig. 3c, and clearly shows that the ZnS NTs have a good contact with the Ag NWs electrodes.

**Figure 4:**
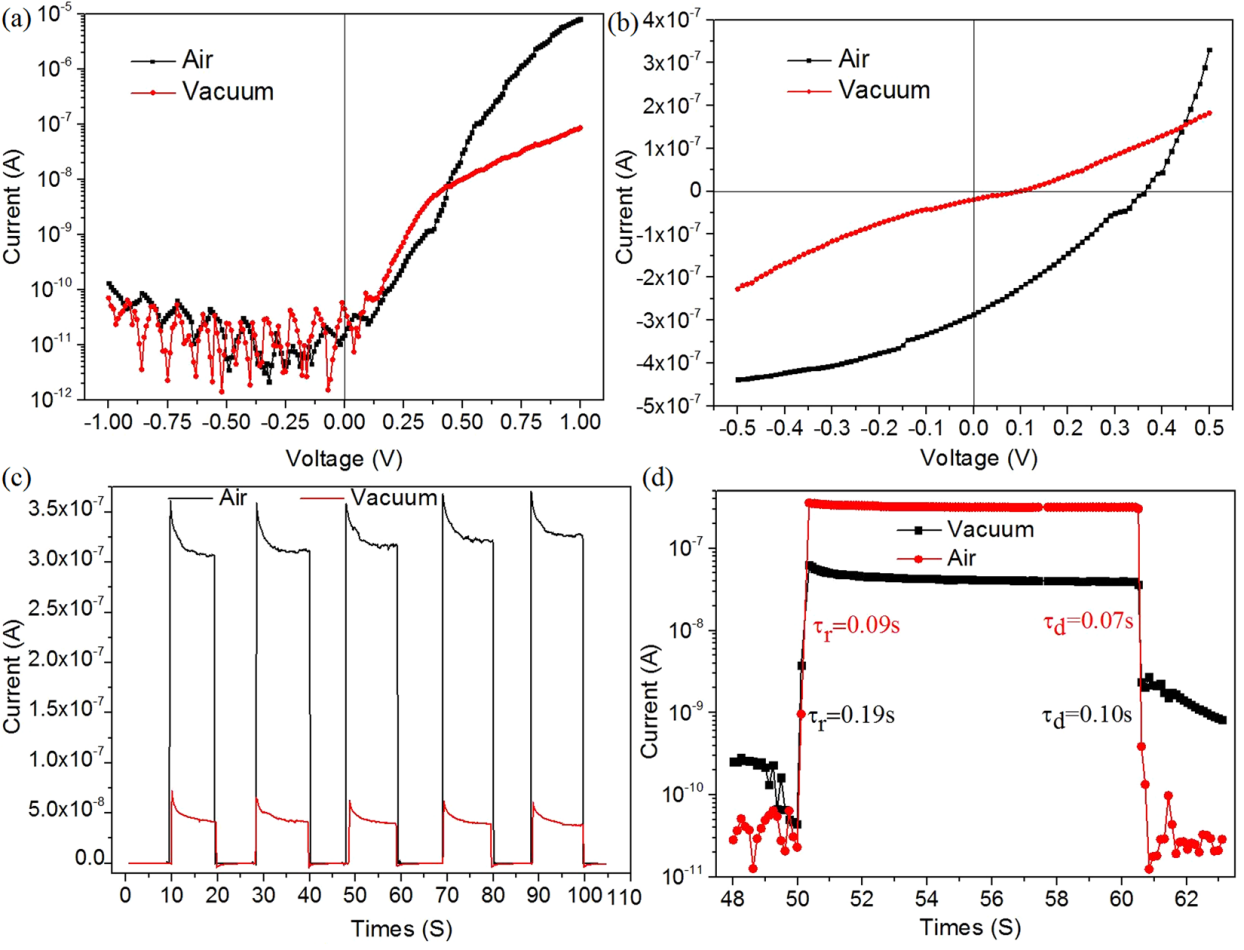
The performance of the nanostructured Ag NWs/ZnS NTs/Ag NWs (MSM) PDs in air and vacuum. The I–V characterizations of the PDs under dark (**a**) and illumination (**b**). (**c**) Short circuit I-t curves of the PDs with the light on/off switching. (**d**) Single on/off cycle of the PDs.

Figure 4a shows typical dark current-voltage (I–V) curves of the nanostructured Ag NWs/ZnS NTs/Ag NWs (MSM) PDs device from −0.5 V to 0.5 V, from which one can clearly observe that the device exhibit an obvious rectifying characteristic, with a rectification ratio of 6.11 × 10^4^ at ±1 V in air. Recently, the rectifier characteristics in metal-semiconductor-metal (M-S-M) junctions with two back-to-back Schottky contacts have attracted a lot of attention. For example, Chiquito *et al*. proposed a general equation to describe the behavior of current flow across a back-to-back Schottky-contacted device^38, 39^.

1 $$J(V)=\frac{{J}_{01}{J}_{02}\,\sinh (\frac{qV}{2{k}_{B}T})}{{J}_{01}\exp (\frac{qV}{2{k}_{B}T})+{J}_{02}\exp (\frac{-qV}{2{k}_{B}T})}$$

2 $${J}_{01,02}={A}^{\ast }{T}^{2}\exp (\frac{q{{\rm{\Phi }}}_{B1,B2}}{{k}_{B}T})$$

where A*, e, k_B_, m_n_* and h is the Richardson constant, elementary charge, Boltzmann constant, effective mass of electron and Planck’s constant, respectively. Here, Φ_B1_, Φ_B2_ is the Schottky barrier height (SBH) value for the M/S and S/M junction, respectively. It has been demonstrated that for a MSM device with same SBHs at two ends, the I–V curve is symmetric, while for a MSM device with disparate SBHs at two ends, the I–V curve is asymmetric. Thus, the asymmetric I–V characteristic presented in this work indicates the SBHs at two ends of the Ag NWs/ZnS NTs/Ag NWs (MSM) devices are disparate. (the disparate SBHs at two ends of the device play key role in the origin of the photoresponse at 0 V in the MSM device, which will be elaborated in details below).

The I–V characteristics under illumination are investigated using xenon lamp (40 mW/cm^2^) as illumination sources. Figure 4b shows the I–V curves of the device under illumination. It is interesting to note that, when illuminated by xenon lamp, such MSM PDs device displays an excellent photovoltaic properties, yielding an open-circuit voltage (V_oc_) of 0.36 V and a short-circuit current (I_sc_) of 2.86 × 10^−7^ A. It is concluded that this device can operate not only at photodiode mode but also at self-powered mode without any external bias.

As an self-powered UV PDs, the photocurrent and dark current of the device operating at zero voltage bias is ≈0.29 μA and ≈14.9 pA, respectively, resulting in the I_on_/I_off_ ratio of up to 19173. For comparison, photodetecting performance parameters of other ZnS nanostructures- and ZnS heterostructure-based PDs are summarized in Table 1. In contrast to ZnS nanobelts- and nanowires-based PDs, the ZnS nanotubes-based PDs have a 1–4 orders of magnitude higher I_on_/I_off_ ratio. Compared with ZnS-ZnO hybrids-, ZnS/SnO_2_ heterostrucuture nanobelts- and ZnS nanobelt/graphene hybrid–based PDs, the ZnS nanotubes-based PDs have a 4 orders of magnitude higher I_on_/I_off_ ratio. The higher I_on_/I_off_ ratio can be ascribed to not only the larger surface-to-volume ratios and hollow tubular architecture of ZnS nanotubes, with which more absorbing light and higher efficiency of charge separation are beneficial to produce larger photocurrent, but also Schottky junction formed between the ZnS NTs and Ag NWs, which effectively hinders the transport of free careers and further leads to a lower dark current.

**Table 1:** Figure of merit of ZnS nanostructure-based PDs and ZnS nanotubes-based self-powered UV PDs.

Photodetector	Light source	Bias(V)	Darkcurrent	Photocurrent	I_on_/I_off_	Rise(s)	Decay(s)	Ref
ZnS nanobelts; Cr/Au	Xenon lamp; 500 W	30	1.0–2.0 pA	643 pA	<643	<0.3	<0.3	30
ZnS nanobelts; Cr/Au	Xenon lamp; 500 W	10	3.0–4.0 pA	1052 pA	<351	<0.3	<0.3	30
ZnS nanobelt; Cr/Au	Xenon lamp; 500 W	20	5 pA	12 pA	2	<0.3	<0.3	31
ZnS nanowires; Ag electrodes	UV light; 1.25 mWcm^−2^	5	0.48 μA	13.5 μA	28	1.8	0.8	32
ZnS-coated ZnO arrays; Au electrodes	Xenon lamp; 150 W	3	15.8 μA	162 μA	10	229	547	33
ZnS/ZnO Biaxial Nanobelt; Cr/Au	UV light; 0.91 mWcm^−2^	5	0.67 μA	4.64 μA		<0.3	1.7	34
ZnS-ZnO Heterostructure; Cr/Au	UV light; 0.2 mWcm^−2^	10	5.3 pA	32.9 pA		0.77	0.73	35
ZnS nanobelt/graphene; Ti/Au	Xenon lamp; 1.25 mWcm^−2^	1	7 μA	37 μA	5	2.8	7.5	36
ZnS nanotubes; Ag nanowires	Xenon lamp; 40 mWcm^−2^	0	14.9 pA	0.29 μA	19173	0.09	0.07	this work

To further determine the response rate and explore the feasibility of the present device for practical application in optical switches, the real-time photocurrent response of the self-powered UV PD to on/off switching optical signal is studied without external power supply. Figure 4c depicts five repeat cycles under on/off switching illumination. It can be seen that the device can be alternatively switched between on- and off-state states with excellent reproducibility and stability. Single cycle of photoresponse is plotted in Fig. 4d. By deducing the rising and falling edges, the rise time (τ_rise_, the time for the current increase from 10% to 90% of the saturation current) and the fall time (τ_fall_, the time for the current decrease from 90% to 10% of its saturation value) is estimated to be 0.09 and 0.07 s, respectively, which are much faster than that of PDs devices based on ZnS nanostructures and ZnS heterostructure at larger bias voltage. Understandably, such a fast response speed could be associated with the hollow tubular architectures of ZnS nanotubes, which significantly influence electron-hole lifetimes and lead to higher efficiency of charge separation. In addition, Schottky junction formed between the ZnS nanotubes and Ag nanowires can further facilitates the effective and rapid separation of the photo-generated carriers, resulting in a fast response speed.

**Figure 5:**
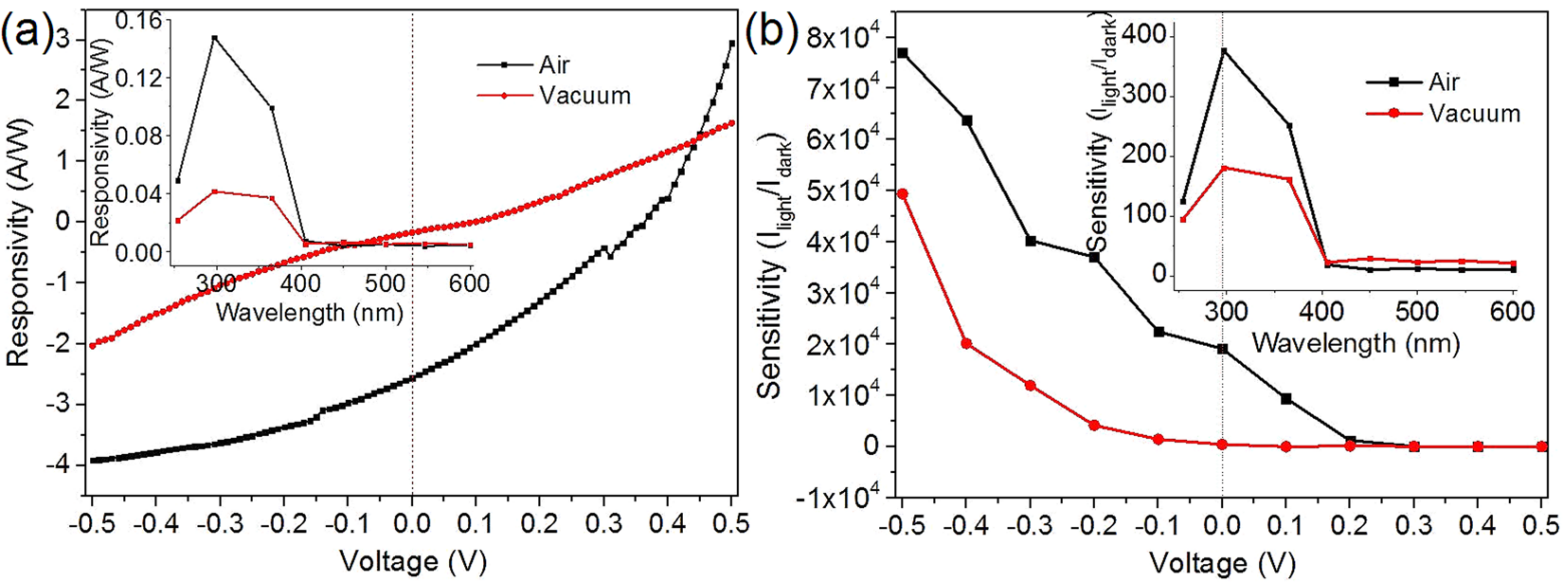
(**a**) Responsivity and (**b**) Sensitivity of the self-powered UV PDs device in air and vacuum. Inset shows (**a**) the responsivity and (**b**) the sensitivity of the device as a function of the incident light wavelength in air and vacuum, respectively.

The performance of photodetector is mainly evaluated by a few critical parameters that include spectral responsivity (**R**_**λ**_), photoconductive gain (**G**), Specific detectivity (D*) and sensitivity (**S**). These four parameters can be expressed using the following equations^40, 41^:

$${R}_{\lambda }=\frac{{I}_{P}}{AP}$$

$$G=[\frac{{\rm{\Delta }}I}{AP\eta }](\frac{h\upsilon }{e})$$

$${D}^{\ast }=\sqrt{A}{R}_{\lambda }/{(2q{I}_{dark})}^{1/2}$$

$$S=\frac{{I}_{p}-{I}_{dark}}{{I}_{dark}}$$

where **I**_**P**_ is the generated photocurrent in the device, **A** is the area of ZnS nanotubes and **P** is the power of the incident illumination, ${\rm{\Delta }}I={I}_{light}-{I}_{dark}$, η is is the effective photocarrier generation efficiency (which is assumed to be 0.7 in our work, when taking the scattering, reflection, and incomplete absorption into consideration), **h** is Planck’s constant, **υ** is the frequency of the incident light, **e** is the elementary charge. At self-powered mode with the bias of 0 V, the calculated values in air of **R**_**λ**_ = 2.56 A/W, **G** = 13.6, D* = 1.67 × 10^10^ cm Hz^1/2^ W^−1^ and **S** = 19172, respectively (Fig. 5).

As the ambient atmosphere dependence of photodetector performance is a critical characteristic for practical applications in unmanned hazardous environments, the Ag NWs/ZnS NTs/Ag NWs MSM self-powered UV PD device is also investigated in different ambient atmospheres. As presented in Fig. 4, photosensing performances of the PDs device in vacuum condition are comparatively studied. The measurements are carried out at room temperature in a vacuum chamber using a mechanic pump as the vacuumizing unit (~1 × 10^−4^ bar). A similar SB-like behavior of the UV detector in vacuum is demonstrated from the dark I–V curve (Fig. 4a), which shows the turn-on voltage and the rectification ratio in vacuum decreases to ~0.31 V and ~1.22 × 10^3^ at ±1 V, respectively. In addition, one can easily note that, at low bias voltages of −0.5 to 0.5 V, the device dark current in air is smaller than that in vacuum, which can be attributed to the the shorter electron lifetime and wider depletion layer at the Ag-ZnS interface as result of the existence of oxygen molecules^42–46^. Figure 4b shows the I–V curves of the device under illumination in vacuum. In comparison with the performance of the device in air, the open-circuit voltage and short-circuit current in vacuum decreases form 0.36 V and 2.86 × 10^−7^ to 0.1 V and 1.92 × 10^−8^ A, respectively. Besides, the real-time photocurrent response of the PD to on/off switching optical signal is studied in vacuum. From the Fig. 4c, it can be observed that a stable output photocurrent also can be generated at zero bias in vacuum, demonstrating that the MSM PDs has potential to UV detect in vacuum condition. The experimental data also clearly indicates that the generated photocurrent in air is almost 8 times larger than that in vacuum, which is in agreement with the ratio of short-circuit current in air and vacuum. Figure 4d shows the calculated response time of PD in vacuum at rising and falling edge are 0.19 s and 0.10 s, respectively. Compared to the response time of PD at rising/falling edge in air (0.09 s/0.07 s), it is observed that the response and recover speed in vacuum are significantly reduced.

In previous research, several mechanisms to explain the response and recovery times of PDs have been proposed based on the oxygen adsorption and desorption process^42–44^. In this work, when turning on the UV light a process of hυ → e^−^ + h^+^ produces larger number of electrons and holes in the ZnS NT and recombine quickly each other, parts of which at the Ag-ZnS interface are separated by the built-in electric field and form photocurrent in the external circuit of the MSM device. In air, oxygen molecules adsorb on the ZnS NTs surface and form oxygen ions by capturing free electrons from the n-type ZnS [O_2_ (g) + e^−^ → O_2_^−^], thereby creating a depletion layer, which leads to the conduction and valence bands are further bent upward and the built-in electric field is enhanced in air, causing higher efficiency of charge separation at the Ag-ZnS interface. In the meantime, the oxygen-joined process, including O_2_^−^ + h^+^ → O_2_ (g) and O_2_ (g) + e^−^ → O_2_^−^, happen on the surface of ZnS and make the photocurrent gradually increase to a saturated value until the desorption and readsorption of O_2_ reach an equilibrium state^45, 46^. In vacuum, although the absence of oxygen molecules speeds the photocurrent quickly increase to a saturated value without the desorption and readsorption of O_2_, the weaker built-in electric field lower the speed of charge separation at the Ag-ZnS interface. Based on the obtained fast response speed at the rising edge in air as shown in Fig. 4d, it is concluded that the response speed of the MSM device is more influenced and dominated by the built-in electric field at the Ag-ZnS interface than the desorption and readsorption of O_2_ occurring on the surface of ZnS. Considering the fact that the oxygen adsorption and desorption rates are significantly decreased in oxygen-deficient environments, the electron lifetime in vacuum will be increased significantly. When turning off the UV light, the increased electron lifetime in vacuum directly lead to current at falling edge reach a minimum value with a slower speed in vacuum than that in air.

Compared with the performance of the self-powered UV detector in air, the responsivity, rensitivity, photoconductive gain and detectivity of the device in vacuum decreases to 0.17 A/W, 435, 0.82 and 7.90 × 10^8^ cmHz^1/2^ W^−1^, respectively (Fig. 5). The results indicate that the photodetecting performance of the self-powered UV detector significantly degenerates in vacuum, which is distinguished from the previous reported ZnO UV PDs that the photocurrent is steeply increased and the photoresponsivity of the ZnO NW detector increases by about 2 orders of magnitude after keeping the sample under vacuum (P < 10^−4^ Torr)^43, 44^.

The inset of Fig. 5a,b shows responsivity and sensitivity spectrum as a function of the incident light wavelength in air and vacuum at bias voltage of 0 V, respectively. A peak responsivity and sensitivity at the wavelength of 297 nm in air and vacuum is observed, revealing that the Ag NWs/ZnS NTs/Ag NWs (MSM) photodetector is highly UV selective whether in air or in vacuum.

**Figure 6:**
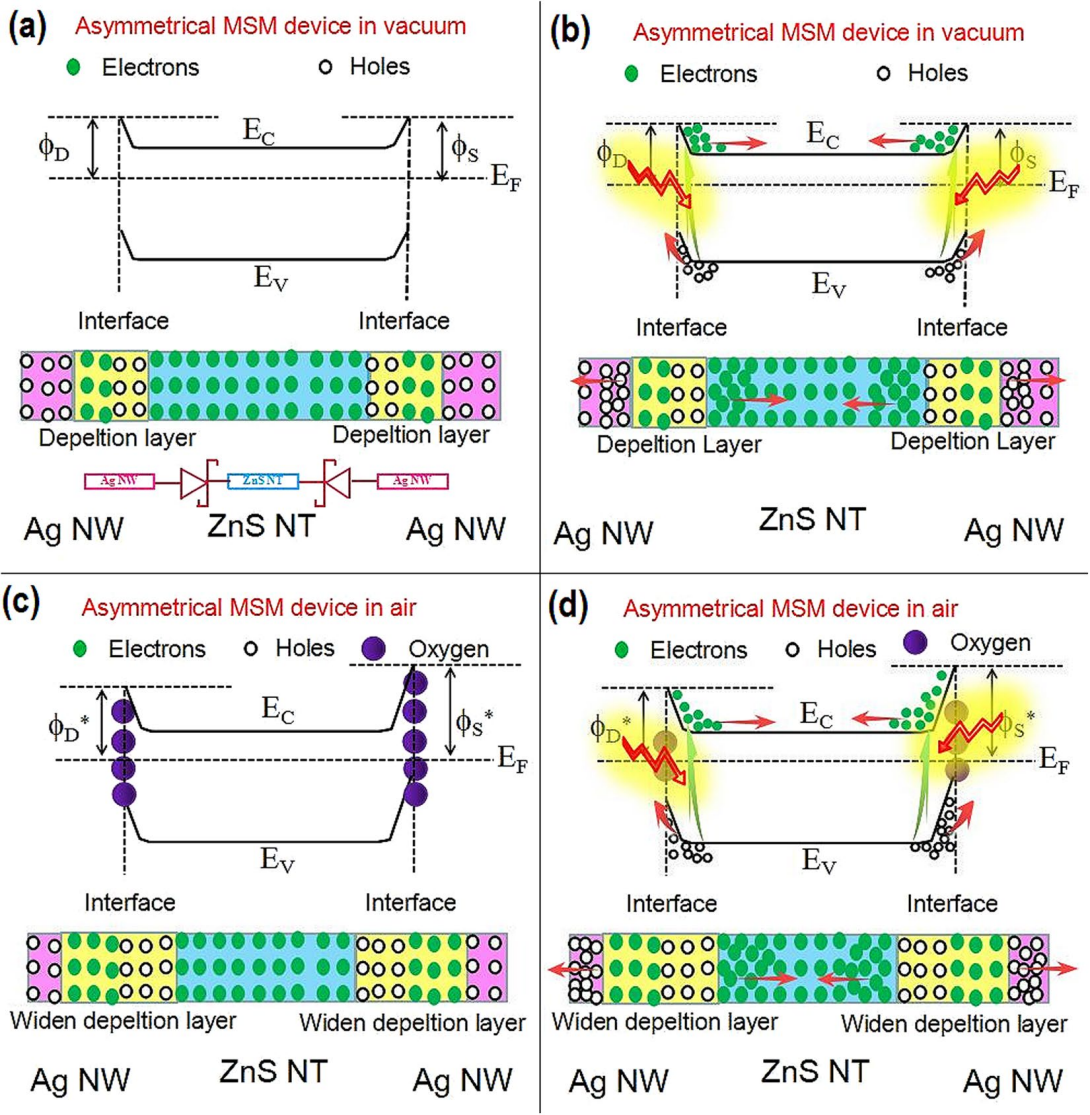
Schematic energy band diagrams of asymmetric Ag NWs/ZnS NTs/Ag NWs (MSM) photodetector under dark (**a**) and illumination (**b**) in vacuum and under dark (**c**) and illumination (**d**) in air to illustrate the working mechanism of self-powered UV PDs based on asymmetric MSM structure. The inset of Fig. 6a shows the circuitry of the back-to-back Schottky device.

The working mechanism of the nanostructured Ag NWs/ZnS NTs/Ag NWs (MSM) self-powered UV PDs is schematically illustrated in Fig. 6 based on energy band theory. The valence band (VB) and conduction band (CB) of the ZnS is −7.7 and −3.9 eV versus vacuum, respectively^47^, and the work function of Ag is 4.26 eV. In this work, when ZnS NTs as photodetecting material connect with metallic Ag NWs electrodes, the electrons will flow from the ZnS NT to the Ag NW until the Fermi levels of Ag (E_F,Ag_) and ZnS (E_F_,_ZnS_) are aligned^20, 46^. Under equilibrium, a Helmholtz double layer will be established at the Ag/ZnS interface, where the Ag NW is negatively charged and the ZnS NT is positively charged near their interface due to electrostatic induction. The electric field induced by the Helmholtz double layer leads to the free charge carrier concentration near the ZnS surface is depleted and forms a depletion layer, which is characterized by excess positive charge. In the meantime, energy bands of ZnS NT bend upward at the interface due to the electric field, the bending degree of the energy band equals the work function difference between Ag and ZnS. The bending of the energy band then forms a Schottky barrier at the interface of Ag NW and ZnS NT (Fig. 6a). Except for the difference in work functions, the Schottky barrier is determined by interface states and adsorption^46^. At the interface of ZnS NT and Ag NW, interface states may exist due to the termination of lattice periodicity at the ZnS NT surface. When compared with the effect induced by the difference in work functions between the ZnS and Ag, the effect of the interface states on Schottky barrier can be negligible.

For the Ag NWs/ZnS NTs/Ag NWs UV PDs device as shown in Fig. 3c, the drain and source Ag NWs electrodes are produced on the same sapphire substrate with the same spin coating progress, thus the drain and source Ag NWs electrodes have the same properties in their thickness, density and resistance. Moreover, owing to the ZnS nanotubes are prepared under the same growth progress, the ZnS nanotubes contacting with the drain and source Ag NWs electrodes have the same crystal quality. The main difference for the MSM device is the contact area of the ZnS NTs with source and drain Ag NWs electrodes, where different numbers of point contacts form between Ag NWs and ZnS NTs.

In ideal vacuum, no gas absorption has effect on the Schottky barrier, which is only determined by the work function of Ag and ZnS. Therefore, the asymmetry Ag NWs/ZnS NTs/Ag NWs MSM device in ideal vacuum would form a symmetry back-to-back Schottky device and is composed of two symmetry Schottky contacts in series, as shown in Fig. 6a. The simplified circuitry of the device is illustrated in the inset of Fig. 6a. Upon illumination, huge amount of electron-hole pairs (hυ → e^−^ + h^+^) appear within ZnS NTs and recombine quickly each other, parts of which at the Ag-ZnS interface are separated by the built-in electric field. As depicted in Fig. 6b, the generated electrons in the conduction band (CB) tend to move away from the contact, while the holes in the valence band (VB) tend to move close to the interface toward the metal side. Due to the identical magnitudes of the Schottky barrier formed at the Drain Ag-ZnS and Source Ag-ZnS interfaces, the two symmetry back-to-back Schottky barriers have the same ability to separate and collect the photo-generated electron and holes, leading to they cancel each other in the external circuit without external power supply.

When the device works in air condition, oxygen molecules adsorb on the ZnS NTs surface and form oxygen ions by capturing free electrons from the n-type ZnS [O_2_ (g) + e^−^ → O_2_^−^], thereby creating a depletion layer with low conductivity near the surface, which leads to the earlier depletion layer is widened and the conduction and valence bands are further bent upward, as depicted in Fig. 6c. For the MSM device, the source Ag-ZnS interfaces provide the larger interface areas for the oxygen molecules adsorption than the drain Ag-ZnS interface. As a result, the width of depletion layer and Schottky barrier height (SBH) (Φ_S_^*^) at source Ag-ZnS interfaces is larger than that at the drain Ag-ZnS interface, as depicted in Fig. 6c.

Upon UV illumination with photon energies above ZnS band gap, electron-hole pairs are generated. Photon-generated holes migrate to the surface and discharge the adsorbed oxygen ions [O_2_^−^ + h^+^ → O_2_ (g)] to photon-desorbed oxygen from the surfaces. The unpaired electrons accumulate gradually with time until desorption and readsorption of O_2_ reach an equilibrium state, resulting in a gradual electron and holes rise until saturation during UV illumination. The two asymmetry back-to-back Schottky barriers differs in ability to separate and collect the photo-generated electron and holes, leading to the formation of photocurrent in the external circuit without external power supply, namely the photovoltaic characteristic.

**Figure 7:**
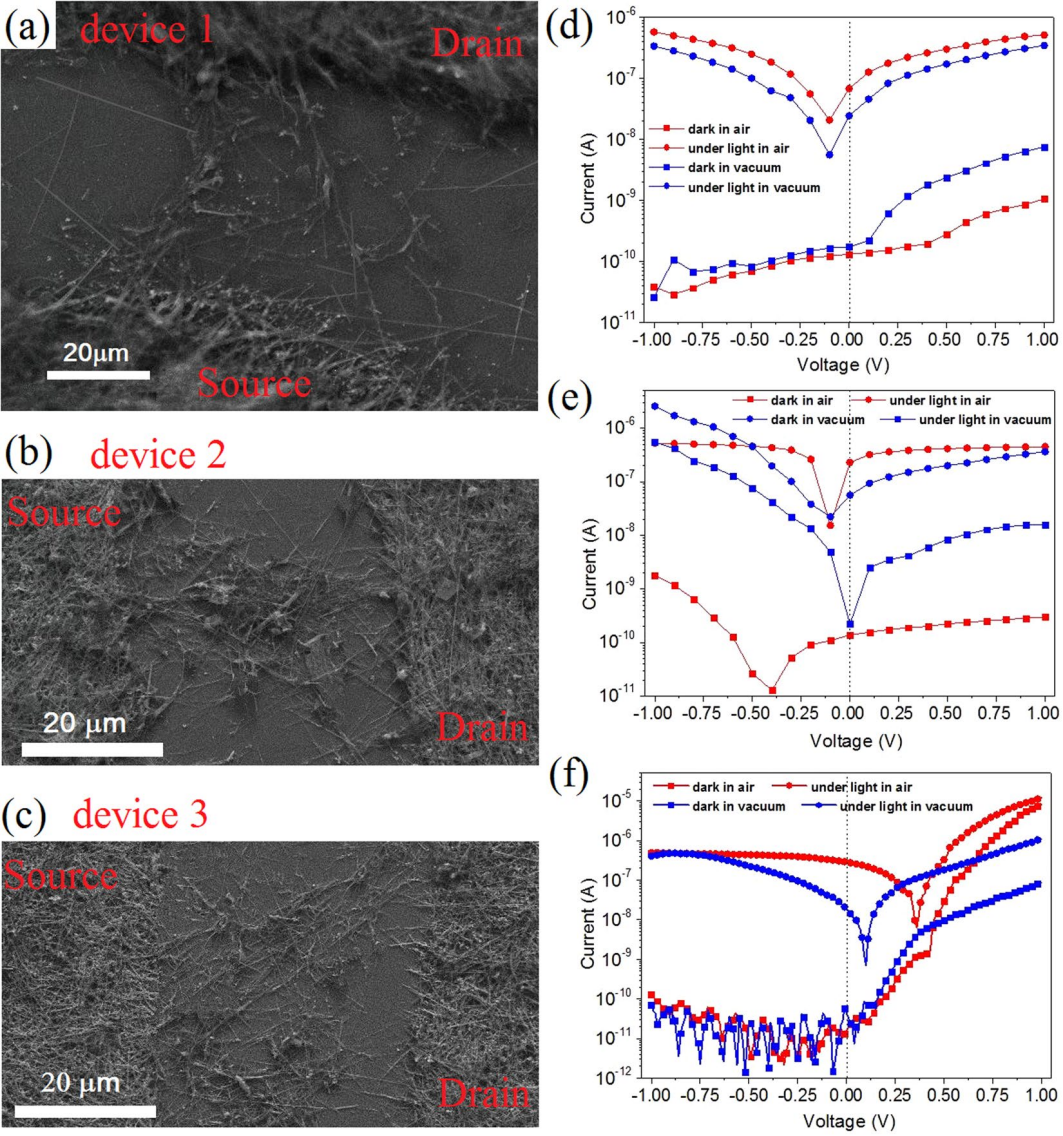
(**a**–**c**) SEM image of the Ag NWs/ZnS NTs/Ag NWs (MSM) self-powered photodetectors device 1–3. (**d**–**f**) Corresponding I–V characteristics of the Ag NWs/ZnS NTs/Ag NWs (MSM) self-powered photodetectors device 1–3 under dark and UV light in air and vacuum.

In order to experimentally validate the photoresponse of the MSM devices at 0 V is dependent on the oxygen absorption and the asymmetrical MSM structure induced by the different contact area of the ZnS NTs with source and drain Ag NWs electrodes, I–V characteristics measurements are carried out on other Ag NWs/ZnS NTs/Ag NWs (MSM) PDs. Figure 7a–c shows the SEM image of the MSM PDs device 1–3, respectively. It is clearly seen that the contact areas of the ZnS NTs with source and drain Ag NWs electrodes are different in the three devices. The contact areas of the ZnS NTs with source (S_s_) and drain (S_d_) Ag NWs electrodes are calculated in the three devices by using the area of single point contact (Ag NW-ZnS NT) multiply the numbers of point contact between ZnS NTs and S/D Ag NWs electrodes, and the results are summarized in Table 2. It is found that the asymmetric ratio of the contact areas of the ZnS NTs with source (S_s_) and drain (S_d_) Ag NWs electrodes (S_s_:S_d_) increase from device 1 to 3.

**Table 2:** Comparison of the structural and characteristic parameters of three Ag NWs/ZnS NTs/Ag NWs (MSM) self-powered PDs in air and vacuum.

Device	Source contact area (mm^2^)	Drain contact area (mm^2^)	Asymmetric ratio	I_on_/I_off_	Ref
1-Air	0.053	0.034	1.6	1.37 × 10^3^	37
1-vacuum				1.41 × 10^2^	
2-Air	0.84	0.36	2.3	1.64 × 10^3^	This work
3-vacuum				2.53 × 10^2^	
3-Air	1.2	0.29	4.1	1.92 × 10^4^	This work
3-vacuum				4.35 × 10^2^	

Figure 7d–f shows the corresponding I–V characteristics of the device 1–3 in air and vacuum. It can be found that all of the MSM PDs exhibit an attractive photovoltaic characteristic. The I_on_/I_of_ values at 0 V of the three devices in air and vacuum are summarized in Table 2. More interestingly, it is clearly seen that with the asymmetric ratio (S_s_:S_d_) increase from device 1 to 3 and the I_on_/I_of_ value is significantly enhanced form device 1 to 3. Moreover, the I_on_/I_of_ values at 0 V of the three devices in air is larger 1~2 orders of magnitude than that in vacuum. These experimental results confirm that the oxygen absorption and the asymmetrical structure play a critical role in the origin of the photovoltaic characteristic.

## Summary

In summary, we have firstly designed and demonstrated a novel type of self-powered UV photodetectors using single-crystalline ZnS nanotubes as the photodetecting layer and Ag NWs transparent network as electrodes. The fabricated self-powered UV photodetectors with asymmetric metal-semiconductor-metal (MSM) structure exhibit attractive photovoltaic characteristics at 0 V bias. Device performance analysis reveals that the as-assembled device exhibits a high on/off ratio of 19173 and a fast response speed (τ_r_ = 0.09 s, τ_f_ = 0.07 s) without any external bias. These values are even higher than that of ZnS nanostructures-based PDs at a large bias voltage. Besides, Its UV sensivity, responsivity and detectivity at self-powered mode can reach as high as 19172, 2.56 A/W and 1.67 × 10^10^ cm Hz^1/2^ W^−1^, respectively. We have also studied the photosensing performance of the self-powered UV PDs in different ambient conditions (e.g., in air and vacuum), showing that the photodetecting performance of the self-powered UV detector significantly degenerates in vacuum. In addition, a physical model based on band energy theory and experimental results is provided to illustrate the work mechanism of the self-driven photoresponse in our device. This study suggests that the self-powered ZnS NTs-based UV PDs will provide a potential approach for the future self-powered optoelectronic devices and integrated systems.

## Methods

### Growth of ZnS nanotubes and Ag nanowires

The ZnS nanotubes were grown on Si substrate via controlled thermal evaporation of ZnS powder (99.999%). A Si substrate rinsed with acetone, alcohol and deionized water was pre-deposited a 8–10 nm Au catalyst layer by RF magnetron sputtering. Then, the Au catalyst layers were annealed in 700 °C for 10 min, to transform them into three-dimensional catalyst islands. The growth substrate for ZnS nanotubes was located 7 cm away from the ZnS powder. And, the quartz boat containing the precursors was placed in the center zone of furnace, where the temperature was rose to 980 °C at a rate of 25 °C min^−1^. Before the reaction, the quartz tube was purged for 20 min with 300 sccm Ar (99.99% purity) and vaccum pump. When the temperature in the furnace reached, the Ar gas as the carrier gas with 200 sccm transport the precursors vapor to the growth substrate. During the whole synthesis process, the pressure in the tube was maintained at 850–950 mbar for 60 min. When the growth was completed, 500 sccm flow of Ar was introduced into the furnace to remove the residual reactants and the sample was were obtained on the substrate after the furnace was naturally cooled down to ambient temperature. In addition, long Ag NWs were prepared by according to the successive microwave-assisted multistep growth (the experiment details of the Ag NWs fabrication were provided in the Experimental Section of ref. 20).

### Device Fabrication and Characterization

To fabricate the Ag nanowires/ZnS nanotubes/Ag nanowires MSM UV photodetector, Ag NWs and ZnS NTs were firstly re-dispersed in ethanol, obtaining the Ag NWs and ZnS NTs being uniformly dispersed in the ethanol. Then, ethanolic Ag NWs were dispersed onto the sapphire substrates by spin coating to produce transparent and conducting Ag NWs networks with uniform distribution and density. The Ag NWs network with a transmittance and sheet resistance of 87% and 64 Ω/sq, respectively, was used as the transparent electrodes of the MSM UV photodetector. To deposit ZnS nanotubes between the drain (D) and source (S) Ag NWs electrodes, we provided a gap of ~40 μm by mechanically scratching the Ag NWs networks using a razor blade. ZnS nanotubes networks were formed between the S/D Ag NWs electrodes through repeated spray coating. To form a good contact and minimize the influence of contact resistance between Ag NW electrodes and ZnS nanotubes, we carried out a fast annealing at 200 °C in Ar atmosphere for 5 min. A xenon lamp (40 mW/cm^2^) with around a 1.5 cm diameter circle illuminated area was used as the light source. Current-voltage (I–V) performance of the photodetector was measured by using Keithley 4200 system. In addition, the photodetector is exposed under UV light for seconds prior to the I–V measurement to eliminate the influence of the surface modification induced by oxygen absorption during the device fabrication.
